# The Enigmas of Tissue Closure: Inspiration from *Drosophila*

**DOI:** 10.3390/cimb46080514

**Published:** 2024-08-09

**Authors:** Xiaoying Huang, Zhongjing Su, Xiao-Jun Xie

**Affiliations:** 1 Department of Histology and Embryology, Shantou University Medical College, Shantou 515041, China; 2 Cancer Research Center, Shantou University Medical College, Shantou 515041, China

**Keywords:** tissue closure, embryogenesis, metamorphosis, oogenesis, wound healing

## Abstract

Hollow structures are essential for development and physiological activity. The construction and maintenance of hollow structures never cease throughout the lives of multicellular animals. Epithelial tissue closure is the main strategy used by living organisms to build hollow structures. The high diversity of hollow structures and the simplicity of their development in *Drosophila* make it an excellent model for the study of hollow structure morphogenesis. In this review, we summarize the tissue closure processes in *Drosophila* that give rise to or maintain hollow structures and highlight the molecular mechanisms and distinct cell biology involved in these processes.

## 1. Introduction

Hollow structure formation is a prevailing phenomenon during animal development. Vital hollow structures include the coeloms and the canals such as the airways, vasculature, excretory and secretory ducts. They either develop to accommodate and protect organs or serve as thoroughfares for the efficient transportation of gasses and liquids into and out of tissues. Although those hollow structures are highly diverse in their architecture and functions, all of them are lined or enclosed by epithelium, suggesting an important role of epithelial tissue in the development and functions of hollow structures.

A variety of strategies are applied by animals to construct hollow structures de novo. Usually, hollow structures can develop directly through the wrapping and budding of epithelium [1]. By the wrapping strategy, edges of epithelia generated by curving, bending, or even cell death fuse to each other and close to form an intact hollow space. In contrast to wrapping, budding occurs when a cluster of cells within an epithelium invaginate and subsequently extend orthogonally to the plane of the original epithelium. Other strategies, including cord hollowing, cell hollowing and cavitation, generate hollow structures in a mass of unpolarized cells by de novo creating polarized epithelium that surrounds a cavity. Tissue closure is only necessary in the wrapping strategy, while it is dispensable for other hollow formation strategies.

As mentioned above, tissue closure is a fundamental step in the formation of hollow structures. Although the physical steps of tissue closure have been well described, the mechanisms driving these events remain incompletely understood. Understanding the genetic programs and biomechanics that drive tissue closure is of great interest because their improper implementation gives rise to disorders such as spinal cord defects including spina bifida and anencephaly, which affect ~1 in 1000 births worldwide [2,3] and represent a major health and economic problem in our society.

Although many studies have helped identify transcription factors, signaling molecules and effectors in tissue closure [4], an important question that remains in the field is exactly how those factors collectively drive tissue fusion at a cellular level. Limited accessibility to the process in humans impedes studies to answer that question. Research in the *Drosophila* system could help us overcome these challenges because of the following reasons: (1) As with all multicellular organisms, *Drosophila* contain a variety of hollow structures, including the dorsal vessels (*Drosophila* heart), wing veins, dorsal appendages, Malpighian tubules and others. They either form during the first several hours of embryogenesis, during metamorphosis or even during wound healing. Many of those processes require tissue closure to finalize the hollow structure. (2) Comparing to higher animals, hollow structures in *Drosophila* usually consist of only a few cell types. (3) Due to the relatively translucent *Drosophila* tissues, such as those in the embryo, and the timeline of tissue closure, live imaging of tissue closure is possible [5]. (4) Many of the developmental mechanisms that *Drosophila* uses during tissue closure are conserved from worms to humans [6].

In this review, we will focus on the tissue closure events throughout the lifespan of *Drosophila,* including embryogenesis, metamorphosis, oogenesis and wound healing. We will highlight the morphogenic details of tissue closure, the underlying molecular mechanisms and the distinct cell biology in those processes.

## 2. General Steps in Tissue Closure

**Figure 1:**
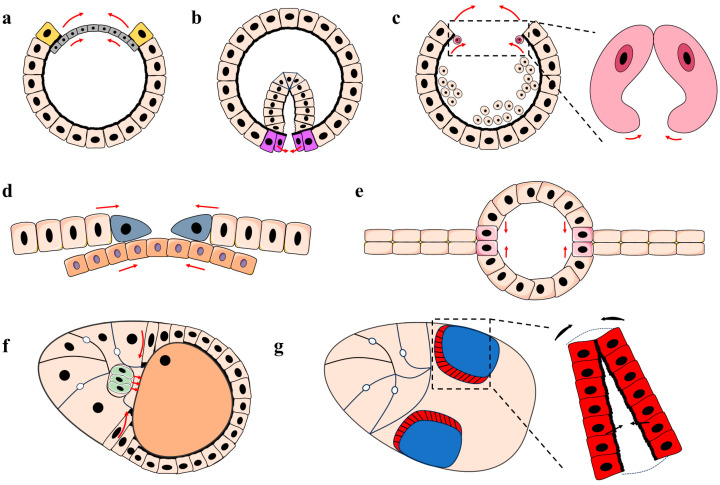
Tissue fusion throughout *Drosophila* development. (**a**–**c**) Schematic cross-sections of a developing embryo. (**a**) Dorsal closure: Large cuboidal cells (tan) represent the ectoderm; the leading edges (yellow) are moving towards each other (red arrows). The amnioserosa is in gray. (**b**) Ventral closure and transient mesoderm tube formation: large cuboidal cells (tan) represent the ectoderm; small cuboidal cells (tan) represent the prospective mesoderm; the leading edges (purple) are moving towards each other (red arrows). (**c**) Dorsal vessel formation: On the left, large cuboidal cells (tan) represent the ectoderm; small round cells (tan) represent the mesoderm; two small round cells (brown) at the dorsal-most region represent the cardioblasts. On the right are two bended cardioblasts, whose dorsal edges have attached to each other and their ventral parts are moving to each other by cell bending (red arrows). (**d**,**e**) Schematic cross-sections of the dorsal thorax (**d**) and wing vein (**e**) during metamorphosis. (**d**) Thoracic closure: Peripodial epithelial cells (tan) on top of the larval epidermal cells (orange) are moving towards each other (red arrows). The leading edges are in gray. (**e**) Wing vein closure: Nucleated cells represent the vein cells, while non-nucleated cells represent the prospective degenerative intervein cells. The leading edges (pink nucleated cells) are moving towards each other (red arrows). (**f**,**g**) Schematic longitudinal section (**f**) and dorsal surface view (**g**) of an egg chamber during oogenesis. (**f**) Follicle cell sheet closure: A large egg (orange) is surrounded by follicle cells (tan, columnar) and nursing cells (tan, large, interconnected by ring canals). Centripetal cells (tan, long) and border cells (green) are moving towards the anterior pole of the egg (red arrows). (**g**) Dorsal appendage formation: On the left are two groups of appendage-producing follicle cells, including the floor cells (red) and roof cells (blue), patterned on the dorsal anterior area of the egg. On the right is the out-of-plane bending followed by a sequence of spatially ordered cell intercalations (black arrows) of floor cells (red).

Tissue closure, no matter what structures or organs it gives rise to, can be distinctly divided into the following five steps: cell fate specification, margin approaching, margin alignment, margin zipping and finally cell sheet rearrangement (Figure 1).

**Figure 2:**
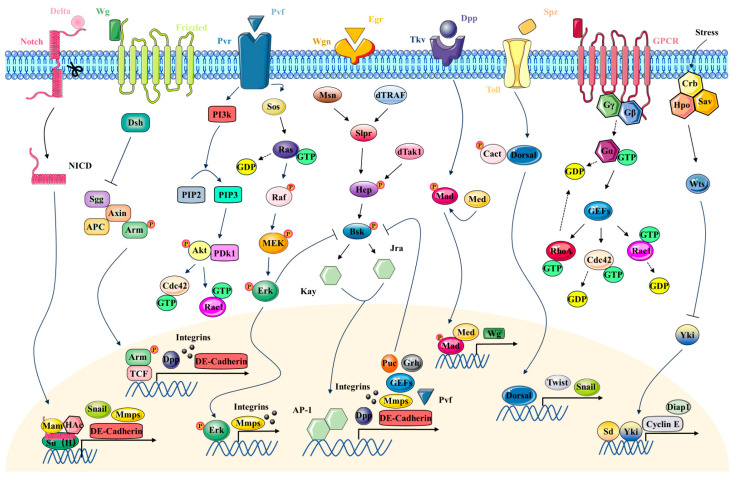
Signaling pathways during tissue closure in *Drosophila*. Notch, Wingless (Wg), Platelet-derived growth factor/vascular endothelial growth factor receptor (Pvr), Jun N-terminal kinase (JNK), Decapentaplegic (Dpp), Toll, G Protein-Coupled Receptor (GPCR) and Hippo (Hpo) signaling pathways are shown.

**Figure 3:**
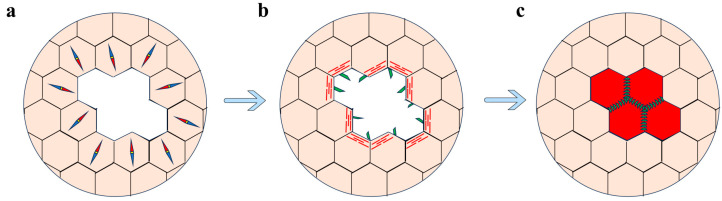
Wound healing in *Drosophila*. (**a**) Initial wound response: epidermal cells (compass) around the wound transit from a quiescent to a motile state. (**b**) Formation of actomyosin cable (red) and cellular protrusions (blue) and migration of epidermal cells around the wound. (**c**) Completion of wound healing: complete covering of the wound by the migrating epithelial cells (red), restoring the integrity of the epidermis.

Firstly, the fate of the epithelium involved in tissue closure is predetermined by the morphogen field. The graded distribution of morphogens such as EGF, BMP and Wnt create the morphogen field, which in turn induces downstream signal transduction (Figure 2). The combination of downstream signaling pathways establishes distinct cell zones in the epithelium with a specific fate in tissue closure. Particularly, free margins of the cell sheet are established and will act as the leading edge for tissue closure (Figure 1 and Figure 3).

Secondly, cell fate choices are translated into cell behaviors such as cell shape change, delamination, migration, extrusion or apoptosis. All these cell behaviors collectively sculpture the cell sheet, causing it to shrink or to bend, which in turn brings the free margins of the cell sheet closer to each other (Figure 1 and Figure 3). The degeneration of cells between the margins (such as the AS in dorsal closure and the larval epithelium in thoracic closure) also contributes to the margin approaching (e.g., Figure 1c). Recently, Balaghi et al. reported that myosin waves promote the alternating contraction of leading and trailing cell edges. Myosin waves and a mechanical asymmetry guide the oscillatory migration of *Drosophila* cardiac progenitors [7].

Thirdly, approaching cell sheet margins align and match each other mainly by cell protrusions and form precise inter-cellular connections between partner cells. Ngan’s work [8] highlights the importance of the coordination between two separate epithelia that are going to fuse. Zhang et al. found that filopodium dynamics are important for precise connectivity between cardioblasts. They concluded that the periodic oscillations of myosin-II mechanically proofread cell–cell connections to ensure robust formation of the cardiac vessel [9].

Fourthly, simultaneously with cell sheet margin alignment, they zip to fuse with each other. The cells with identical positional identities in the margin recognize each other and build up new adhesion sites between them. Actin-mediated protrusion shortening was hypothesized to generate the zipping force [10].

Finally, the newly formed cell sheet will be rearranged by cell extrusion, neighbor exchanges or other ways to ensure the formation and maintenance of regular cell packing and relieve mechanical stresses in the tissue [11].

## 3. Tissue Closure in Embryogenesis

The fertilized egg of *Drosophila* undergoes 13 rounds of synchronous and rapid nuclear division to form a syncytium with approximately 6000 nuclei. During the 14th division cycle, the cellularization of the cortical nuclei generates the blastoderm epithelium that encloses the whole embryo [12], which can be considered the first tubular structure in embryogenesis. Collective cell activities such as migration, invagination and fusion in the blastoderm epithelium give rise to the ectoderm, mesoderm and endoderm, which will orchestrate to make complex tissues and organs. Many more tubular structures are generated during this process, including the transient mesoderm tube, dorsal vessel, digestive tract, trachea, etc. We will discuss the morphogenesis of tubular structures, which are formed by the wrapping and fusion of epithelia in embryogenesis.

### 3.1. Dorsal Closure

Dorsal closure (DC) in *Drosophila* embryonic development is a highly orchestrated morphogenetic process involving the coordinated movement and rearrangement of epithelial cells [13,14,15,16,17,18,19,20,21]. During stage 13 of embryonic development, the embryo’s germ band extends anteriorly by approximately 65–70% of the embryonic length and subsequently retracts posteriorly. Germ band retraction leaves a dorsal opening covered by an extraembryonic tissue, the amnioserosa (AS), which accounts for about 40% of the embryonic circumference [13,19]. As the retraction concludes, the DC commences (Figure 1a). Initially, the lateral epidermal (LE) cells from opposing sides of the ectoderm become polarized, and the leading edges are formed (Figure 1a). After that, the LEs migrate towards the dorsal midline, and the filopodia and lamellipodia extend from the leading edges. Finally, the opposing LE cells meet at the dorsal midline and form new adherens junctions to close the dorsal opening [13,14,15,16,19,21,22].

The AS is a crucial epithelial tissue formed by the specialization of the dorsal-most cells of the embryo. This tissue plays a vital role in providing an additional pulling force to drive the LE cells toward the midline through contraction and cell apoptosis during DC [13,15,20,21]. Both the degeneration of AS, the contraction of single AS cells and the contraction of a supracellular actomyosin cable, the so-called “purse-string”, in the leading edges generate the mechanical force to pull the epithelial sheets towards the dorsal midline. Interestingly, AS cells exhibit pulsed apical constriction governed by repetitively appearing F-actin/Myosin II foci [13,15,17,21]. Additionally, they reduce their circumference and total area via endocytosis, which facilitates the clearance of connective materials and membranes [13,18]. Furthermore, AS cells modulate adhesion among dorsal epidermal cells through the secretion of proteins such as Innexin 3, which form gap junctions to facilitate the migration of dorsal epidermal cells [23]. Eventually, AS cells undergo invagination and programmed cell death during the final stages of DC. These processes ensure that the AS tissue provides the necessary pulling forces and rearrangements of the appropriate cells to facilitate the fusion of the dorsal epidermis [14,15,24].

The precise modulation of cellular activities and intercellular dynamics is essential for successful DC [16,19,24]. This procedure, including cellular and tissue tension, contraction and morphological changes, involves several signaling pathways and molecular regulators (Figure 2) [13,15,16,19,21,22,25,26]. The Jun-terminal kinase (JNK) signaling pathway is pivotal for activating transcription factors that regulate cytoskeletal reorganization and cell migration [13,19,22,27,28]. A multitude of studies have shown that biomechanical stress and strain endured by LE cells activate the JNK homolog Basket (Bsk) [13,16,19]. This activation phosphorylates the transcription factors kay and Jra, which dimerize to form the AP-1 transcription factor complex. Then, the complex induces the expression of target genes related to Dpp and puckered (puc), which subsequently regulate cell motility and cytoskeleton [16,19]. Dpp signaling is another key pathway in DC. Disruption of this pathway results in a failed closure. In addition, Dpp determines the fate and differentiation trajectory of dorsal cells via the induction of transcription factors such as hindsight and u-shaped [16]. Furthermore, mutations in the key components of the Wingless (Wg) signaling cascade, such as Dishevelled (Dsh) and Armadillo (Arm), result in defects in dorsal midline closure, underscoring the strong association between DC and Wg signaling [19,25]. Studies have highlighted the critical function of Wg signaling in regulating the expression of the Dpp gene in LE cells [19]. However, mutations in Fos or Kay reduce the efficacy of Wg signal transduction, suggesting that Wg pathway activation is intricately dependent on JNK signaling [19,25]. Additionally, new findings indicate that Pvr mutations lead to incomplete AS internalization and abnormal epidermal zippering, implicating that alterations in Pvr levels significantly impact the structure and function of tissues involved in closure [29].

### 3.2. Ventral Closure and Transient Mesoderm Tube Formation

The transformation of a single-layered blastoderm into a multilayered gastrula is called gastrulation. At the beginning of gastrulation, the collective movement of cells creates furrows in the blastoderm [30]. Some of them, such as the cephalic furrow and dorsal transverse furrows, are transient, and eventually cells in those furrows return to the surface [31]. However, a large anterior-to-posterior furrow in the ventral domain, the ventral furrow, is formed to pinch the prospective mesoderm cells off into the interior of the embryo (Figure 1b) [30].

The morphogen Spätzle pre-determines the ventral furrow domain in blastoderm [32]. Spätzle binds to the Toll receptor and induces nuclear translocation of transcription factor Dorsal (NF-κB), which further activates two important transcriptional factors, Twist and Snail (Figure 2) [33]. Factors downstream of Twist and Snail synergistically control the cyclical activation and inactivation of small GTPase RhoA [32]. The pulsing activity of RhoA activates the pulsing constriction of actomyosin on the apical surface of the presumptive ventral furrow domain [34]. Apical constriction shrinks the outer surface of the epithelium, resulting in cell shortening, invagination and thus ventral furrow formation [30]. As the ventral furrow deepens, the lateral edges of the furrow become closer and fuse with each other. Cells in the ventral furrow pinch off and form a transient epithelial tube inside the embryo, which then gives rise to the mesoderm germ layer. At the same time, the cells flanking the ventral furrow fuse and seal the ectoderm [32]. There are two closure events in this process: one is the closure of the ventral furrow, and the other is the closure of the ectoderm to seal the gap generated by the invagination of the ventral furrow.

### 3.3. Dorsal Vessel Closure

The dorsal vessel, the heart of *Drosophila*, is a simple linear tube that resembles the vertebrate heart at an early stage of development. The morphogenesis of the dorsal vessel during *Drosophila* embryonic development also exemplifies the classic process of tissue closure. Shortly after gastrulation, the mesodermal cell mass spreads laterally and arranges itself into a monolayer beneath the ectoderm. Mesodermal cells that migrate to the most dorsal region of the embryo develop into cardiac progenitor cells and then differentiate into cardioblasts and pericardial cells [35,36]. During the process of DC, the cardiac progenitor cells from either side of the embryo approach each other at the dorsal midline. Those cells first establish dorsal contacts with the contralateral counterpart and then undergo cell shape changes to make ventrally contacts, resulting in the formation of a continuous heart tube along the anterior–posterior axis (Figure 1c) [35,36,37,38].

The formation of the dorsal vessel in *Drosophila* is influenced by several key proteins and signaling pathways. Tinman, a homeobox transcription factor, led to the identification of its mammalian homologue, Nkx2.5, as the first essential factor for the specification of the cardiogenic region in both flies and vertebrates [36,39]. Tinman promotes cardiac cell differentiation and morphogenesis by regulating the expression of a series of genes, including those for cardiac structural proteins and proteins involved in cardiac contraction [35,40]. Furthermore, Tinman can interact with other transcription factors and signaling pathways, such as the Dpp and Wg pathways, to induce differentiation of dorsal-most mesodermal cells into cardiac precursors [35,39]. In mutants lacking Heartless and Tinman, the dorsal mesodermal cells fail to receive the inductive signals from the Dpp pathway, which impede the dorsal spreading of the mesoderm and block the formation of the heart and other visceral tissues [35,39,40]. Additionally, FGF signaling through the Heartless receptor and its ligands Pyramus and Thisbe is essential for the dorsal migration of mesodermal cells and the proper formation of cardiac mesoderm. Mutations in Frizzled, the receptor for Wg, result in the failure of cardiac precursor cell formation in *Drosophila* embryos. Moreover, Epidermal Growth Factor Receptor (EGFR) signaling is also crucial for the differentiation and maintenance of cardiac mesoderm morphology. Overexpression of EGFR in the mesoderm leads to an increased number of cardiac cells, highlighting its role in the proliferation and/or maintenance of cardiac cells [40]. The regulatory and integrative mechanisms of these signaling pathways in *Drosophila* are highly conserved in vertebrates, providing a foundation for understanding the genetic regulation of heart development in vertebrates [36,39,40].

## 4. Tissue Closure in Metamorphosis

Metamorphosis is a post-embryonic developmental process that results in a spectacular morphogenic transition between the vegetative and sexually reproductive stages of a life history [41]. In holometabolous insects such as *Drosophila*, which undergo complete metamorphosis, adult structures such as the appendages and abdominal epidermis are developed and transformed from their corresponding miniature precursors found in the larvae, known as imaginal disks and histoblast nests. The imaginal disks will give rise to all the adult appendages, including wings, eyes, legs, etc., while the histoblast nests will give rise to the adult abdomen and internal organs such as the midgut and salivary gland [42]. Similar to embryogenesis, many tubular organs are formed during metamorphosis. Here, we will focus on the processes of thoracic closure and wing vein formation to illustrate tissue closure during *Drosophila* metamorphosis.

### 4.1. Thoracic Closure

During the pupal stage, imaginal disks—epithelial sacs affixed to the inside of the larval epidermis—embark on a complex journey of cellular differentiation and morphogenesis. These processes lead to the formation of the fly’s external structures, such as wings, eyes and appendages [43]. Among these, the largest pair of imaginal disks are wing disks, which undergo a series of cellular proliferations and tissue augmentations to form the fly wings and thorax (notum) [44]. The adult thorax emerges as a bilaterally symmetrical structure, formed by the fusion of identical heminotal epithelia (HE) derived from the proximal regions of both wing disks in a process also known as thoracic closure [44,45,46]. The eversion of the heminotal epithelium is crucial for the formation of the notum [43,45,47,48,49], which is developed from flat, non-polar squamous peripodial epithelium (PE) on the lateral side and columnar proper epithelium on the medial side [24,43,48,49,50].

In the early stages of pupal development, the outer basal surface of the disk epithelium invades the inner basal surface of the larval epidermis. This invasion triggers an epithelial–mesenchymal transition in segments of the PE, leading to the rupture of the basement membranes of the opposing PE and larval epidermal cells (LEC) [43,48]. At this point, each HE is everted [43,45]. Select PE cells then form the leading edge of an epithelial sheet, initiating a movement toward the dorsal midline and replacing the intervening larval epidermal tissue [43,48,49]. The proliferation of the disk proper cells, along with the contraction of the LEC near the midline, facilitates the migration and fusion of the HE (Figure 1d) [46]. This migration continues until the advancing HE meets the contralateral HE’s leading edge. Through the reestablishment of cell–cell junctions, they merge to form a new epithelium at the midline of the future adult body. This new epithelium will eventually become the epidermis of the adult thorax [43,46,48,49,50]. Integrin-dependent anchorage between the LEC layer and the flanking heminotal epithelia is crucial for relaying contraction forces from the former to the latter during thorax closure [46].

Thoracic closure is an important event in the maturation of *Drosophila* pupae, which involves intricate mechanisms of epithelial structure destruction, cell migration and tissue remodeling [43,48]. In essence, this is a developmentally induced wound closure, that results in the formation of new epidermis in adults [48]. Thorax closure shares similarities with embryonic DC in terms of cellular migration, morphological changes and signaling pathways [24,29,50]. For instance, the LEC layer functions during thoracic closure similarly to AS in DC by generating propulsive force through non-muscle myosin II-mediated contractions, which promote the convergent migration and fusion of epidermal borders toward the midline [43,46]. Furthermore, as the epithelial sheets on both sides zip together, the space between cells decreases, and the LECs, like the AS cells, undergo apoptosis and are cleared from the cell layer. This helps prevent cell overcrowding and promotes further contraction of the cell layer [43,46]. The signaling pathways involved in seamless epithelial fusion, such as Dpp and Wg, typically act in coordination with or under the regulation of the JNK signaling pathway [29,44,49,50,51,52]. However, the role of Pvf1/Pvr in tissue closure is independent of JNK signaling [29]. Studies have shown that excessive activation of Pvr leads to increased accumulation of membrane PIP3, suggesting the involvement of PI3K signaling (Figure 2) [29].

### 4.2. Wing Vein Closure

The *Drosophila* adult wing blade is a flat, translucent airfoil with five main longitudinal veins (LVs) and two main cross veins (CVs) [49]. Those veins subdivide the wing blade into several intervein regions. While the intervein cells undergo apoptosis shortly after eclosion and leave their lifeless cuticle in situ in the mature wing, the cells along the veins are alive [53]. The veins, as a hollow tubular structure wrapped by a single layer of epithelium (Figure 1e), serve as hydraulic pipes for wing expansion [54], as spars for flapping flight [55], and as conduits for hemolymph, nerves, and tracheae [56] in *Drosophila*.

The developmental mechanisms of *Drosophila* wing vein have been subjected to intense studies and are well established. The wing imaginal disk initially appears as a cluster of about 25–30 cells around embryonic stages 12–13 [57], which invaginates to form small epithelial sacs at embryonic stage 14 [49]. This single-layered epithelial sac grows exponentially through three continuous larval stages to form a mature larval wing disk of about 35,000 cells [49]. At the late-third-instar stage, the patterns of longitudinal provein zones are established, as indicated by the monitoring of gene expression that both respond to or potentiate EGFR signaling. However, the tubular structure of the vein cannot form until metamorphosis. During metamorphosis, the single-layered wing disk undergoes eversion to become an adult wing composed of two epithelial cell layers. The LV proveins that are formed separately on the prospective dorsal and ventral surfaces of the wing imaginal disk align to make the hollow tubule structure of the mature veins (Figure 1e). Subsequently, the cuticles of the intervein regions become tightly bonded to form a flexible wing blade, while the cuticles of the vein regions form tubes, lined by living cells, through which hemolymph circulates in mature adult insects.

The formation of wing veins requires the accurate differentiation of vein and intervein cells. At least five different signaling pathways converge to position and maintain vein and intervein territories in the wing: Hedgehog (Hh) signaling, bone morphogenetic protein (BMP) signaling, EGFR signaling, signaling mediated by the *Drosophila* Wnt/Wg and Notch signaling mediated by its ligands, Delta and Serrate (Figure 2) [58]. Another important property of vein cells is that no connections exist between the dorsal and the corresponding apposed ventral cells, while there are connections between the dorsal and ventral intervein cells at their basal sides. Those connections are vital for the reapposition of the dorsal and ventral epithelia of the wing blade.

## 5. Tissue Closure in Oogenesis

The *Drosophila* eggshell has three tubular chorion structures: the eggshell itself, the dorsal appendages (respiratory filaments) for embryo respiration and the cone-shaped micropyle that provides the entry channel for the sperm to fertilize the egg. All these structures are synthesized by corresponding follicle epithelial tubes. For micropyle, its corresponding follicle epithelial tube is generated by epithelial budding [59]. However, the follicle epithelial tube that builds eggshells and appendages is formed by epithelial wrapping and is thus discussed blow.

### 5.1. Follicle Cell Sheet Closure

*Drosophila* eggs develop in the egg chamber. At its earliest stage (oogenesis stage 1) [60], the egg chamber is composed of germ line cyst cells encapsulated by a somatic follicle epithelium. In the following oogenesis stages, one of the cyst cells becomes an oocyte, which is localized at the posterior of the chamber, and all the other cyst cells become nurse cells to nourish the oocyte. Thus, in the early stages (oogenesis stages 1–9), the anterior surface of the developing oocyte contacts directly with the nurse cells but not the follicle cells, while the other part of oocyte is covered by columnar follicle cells. During stage 9, some of the follicle cells, the so-called border cells and centripetal cells, which used to be in contact with the nurse cells, migrate to contact the anterior surface of the oocyte (Figure 1f) [61]. They, together with those columnar follicle cells that have been coating the oocyte, form a complete cell sheet to enclose the oocyte during stage 10 [59]. The migration of centripetal cells resembles a closing diaphragm [61], while the border cells act like a cellular “patch” to fill the hole in the center of the diaphragm.

Elegant studies have identified signaling systems important for border cell delamination, migration, and neolamination, including JAK/STAT [62], Notch [63], PDGF/VEGF receptor (PVR) [64] and EGFR pathways [65]. Recently, Miao in Montell’s laboratory demonstrated a channel-independent role of gap junction proteins (Innexins) in establishing contacts between border cells and centripetal cells to form the continuous follicle cell sheet that envelops the oocyte [61]. Their results also highlight the importance of innexin-regulated microtubule dynamics in this process.

Centripetal cell migration is fundamentally different from border cell migration. It has superficial similarities to convergent extension [66,67]. However, similar to border cells, centripetal cell migration is regulated by a similar set of signaling pathways, including Dpp [68], Notch [69] and EGFR [68]. Interestingly, the adherens protein E-cadherin and the mechanoresponsive protein H-spectrin have impacts on the site of centripetal cell migration [67,70].

### 5.2. Dorsal Appendage Formation

The dorsal appendages are two prominent tubular extensions of the chorion at the anterior egg chamber that facilitate gas exchange [71]. Gurken (Grk, EGF signaling pathway) and Decapentaplegic (Dpp, BMP signaling pathway) intersect to specify two patches of follicle cells in the dorso-anterior region, which are the primordia of the dorsal appendages (Figure 1g) [61]. Each of the two primordia contains approximately 70 cells and has two adjacent subdomains, a hinge-shaped row of approximately 15 “floor” cells bordering a patch of approximately 55 “roof” cells [71]. Interestingly, regardless of how the EGF or BMP pathways are manipulated, the follicle cells always generate a row of floor cells adjacent to a group of roof cells, suggesting the development of floor and roof cells is extremely robust. At stage 11, the constriction of the apices of the roof cells causes the epithelium to bend outward. Simultaneously, the adjacent lateral floor cells converge towards the midline, elongate and extend beneath the roof cells, zipping their apices to seal off the tube (Figure 1g) [72].

## 6. Tissue Closure in Wound Healing

Numerous studies have demonstrated the effectiveness of *Drosophila* as a model organism for studying wound healing across all developmental stages, from embryonic to adult [24,27,73,74,75,76,77,78]. Because of the difficulty in analyzing the cellular and molecular mechanisms of re-epithelialization in human tissues, the *Drosophila* model allows researchers to explore the dynamic changes and regulatory paradigms of wound healing across various physiological and developmental contexts, simulating tissue repair processes observed in mammals [24,27,74,77,79]. The imaginal disks of larvae, in particular, serve as a model for regeneration studies. Injured imaginal disks exhibit significant regeneration capacity and distinct pattern features, and their conserved genes and signaling pathways highlight the importance of epigenetics in regulating regeneration [49,74,79,80].

### 6.1. Characteristics of Wound Healing across Developmental Stages

Throughout the various developmental stages of flies (embryo, larva, pupa and adult), the wound healing process exhibits both similarities and distinct features [24,76,78]. Wound healing at the embryonic stage primarily relies on cell migration and morphological changes to achieve re-epithelialization (Figure 3) [77,81]. After injury, epidermal cells around the wound site transit from a quiescent to a motile state. These cells re-establish polarity, specifically planar cell polarity and apical-basal polarity, to guide the directionality of cell migration [77]. Similar to DC, wound closure depends on the contraction of actomyosin “purse string” surrounding the wound site. The “purse string” propels the epidermal cell movement to cover the wound gap through the interdigitation of actin protrusions [77,81,82,83,84].

The wound healing in *Drosophila* larvae and adults involves intricate immune responses. However, immune responses in wound healing are relatively weak at the embryonic and pupal stages. Following puncture injury, specialized hemolymph cells such as crystal cells and lamellocytes are recruited near the wound site and play an essential role in healing [27,76,85,86,87,88]. Crystal cells facilitate scab formation, with their granule-deposited crystals forming emboli that eventually turn into a melanic scab [27,76,86]. The initial stages of wound healing involve the fusion of epidermal cells surrounding the embolus, creating a syncytium that expedites wound coverage [27,89,90,91]. In adults, the process becomes more complex due to the robustness of the adult cuticle, involving not only cell migration and proliferation but also the re-establishment of the structural integrity of the epidermis [91].

### 6.2. Signaling Pathways in Wound Healing

Within hours post-injury, the expression of Jra, Kay and puc (negative regulators of the JNK pathway) is detectable at the wound edge [73,76,89,91], while ectopic expression of Wg is observable at the injury site of imaginal disks [79]. This finding highlights the critical role of JNK and Wg signaling in developmental closure events, as well as wound closure. Recent studies have revealed that the increased dual oxidase activity generates abundant reactive oxidative species (ROS) after epidermal tissue injury, which stimulates an inflammatory response and cell migration around the wound, thereby promoting wound healing [73,78]. ROS, via the Src42A kinase, promotes the polarization of DE-cadherin and myosin around the wound [48]. It also activates the transcription of target genes in the Grainy head (Grh) signaling pathway and participates in the reconstruction of the epidermal barrier [73].

Loss of JAK/STAT signaling inhibits cell–cell fusion, as observed in transgenic flies using STAT activation reporter genes, suggesting that syncytia formation in the wound region is regulated by the JAK/STAT pathway [89]. Additionally, Pvf1/Pvr are essential for epidermal wound closure [29,52,85,92]. Deficiency of Pvf1/Pvr hinders wound healing [76]. The activation of the transcription factors yorkie (Yki) and Scalloped (Sd) within the Hippo pathway also affects actin aggregation around the wound edge and wound closure (Figure 2) [27,93]. The synergistic interplay of these pathways promotes epidermal cell migration, fusion, polarization and reconstitution of the epithelial barrier, effectively sealing the wound and restoring tissue integrity.

## 7. Common Strategies in Tissue Closure

A series of cellular processes and their collective behaviors are involved in tissue closure. As we discussed above, although tissue closure may lead to the construction of different structures and organs, they utilize certain common strategies. We will discuss those common strategies in the following sections.

### 7.1. Signaling for Tissue Closure

Before the initiation of tissue closure, the cell fate must be allocated, and at the same time, the cell sheet must be patterned. Cell signaling is important to the patterning of the cell sheet for tissue closure (Table 1). Evident examples include specific cell sheet pattern formation in the wing vein and dorsal appendage formation (Figure 1g). The gradients of morphogens such as DPP and Wnt, which generate morphogen fields at the tissue level, play central roles in the patterning of the cell sheet. The final effect of the morphogen field is precise and robust. It can define the cell fate with the resolution of a single row of cells, as seen in the roof cells in dorsal appendage morphogenesis and other tissue closure processes (Figure 1). It also generates the same pattern repeatedly, generation by generation. Local morphogens are equally important. Johansen et al. found that cell intercalations are oriented circumferentially due to a gradient of JAK/STAT pathway activation, which is essential to achieving proper elongation [94].

The most active signaling site is the margins of cell sheets, the leading edge of tissue closure, in which cells energetically interact with the environment and translate environmental cues into cell behaviors. The formation of highly dynamic filopodia in margin cells seems to be a common phenomenon in tissue closure, indicating bustling signal transduction at the leading edge. As seen in dorsal vessel closure, differential filopodia adhesions, together with periodic contractions generated by Myosin II oscillations, proofread cell–cell connections between cardioblast cells, ensuring precise cell matching and robust dorsal vessel formation [9].

Mechanosignaling is applied in tissue closure to orchestrate cell behaviors to create distinct structures. Several cellular compartments are involved in mechanosignaling, including the plasma membrane, cytoskeleton, nucleus, and other organelles [95]. The adhesion complexes in the plasma membrane can convert mechanical forces into biochemical signals to influence cell physiology [95]. Take the DC as an example. A feedback loop between cell adhesion and intracellular signaling pathways has been observed in the leading edge. Karkali et al. found that integrin-mediated anchorage could modulate JNK activity, and in reverse, JNK activity is necessary for the assembly of filopodia and sustains the adhesion between the AS and the lateral epidermis during DC [96]. For more details on mechanosignaling, we recommend recent reviews [11,95,97].

### 7.2. Forces for Tissue Closure

At the tissue level, the organism employs a variety of strategies to bring two opposing margins closer together. Firstly, the most prevalent strategy is the collective migration of cell sheets. As observed in processes such as DC, dorsal vessel formation, thoracic closure and follicle cell sheet closure (Figure 1), cell migration enables the boundaries of the cell sheet to approach each other, gradually reducing the distance between the leading edges. Secondly, the contraction of a cell sheet located between two margins, such as AS in DC, the larval epidermis in thoracic closure and the roof cells in dorsal appendage formation (Figure 1), can indeed generate forces to mediate tissue closure [46]. Similarly, the bending of cell sheets or cells can also bring cell boundaries closer together, promoting tissue fusion, as shown in ventral furrow formation and dorsal vessel closure (Figure 1b, c). Ultimately, the converging cell boundaries will engage with each other through protrusions such as filopodia and lamellipodia, establishing robust cell adherens to the complete closure of the tissue.

At the cellular level, forces may be generated, guided and resisted by cells, the extracellular matrix, interstitial fluids and the ways they are organized within the tissue [98]. The skillful application of force enables morphogenesis, including tissue closure. The molecular machinery of force utilization at the cellular level is exceptionally complex, with the spatio-temporal control of actomyosin and cell adhesion systems being the most important aspect. Actomyosin-driven mechanisms are highly conserved throughout evolution [97]. An apical meshwork of diametrical actomyosin fibers plays the predominant role in constricting the apical area, which is key to cell sheet folding [99,100]. Balaghi et al. found that dynamic actomyosin waves promote cardioblast migration in dorsal vessel formation in *Drosophila* [7]. Interestingly, the actomyosin and cell adhesion that span across multiple cells form a supracellular contractible actomyosin cable, namely the purse-string, playing a crucial role in tissue closure [83]. Other mechanisms for regulating mechanics at the cellular level include syncytium formation, neighbor exchange, cell growth and cell deletion, etc. [11,95,97]. Although not reported in tissue closure, endocytosis and exocytosis to regulate cell membrane tension may also apply to tissue closure [101]. Multiple mechanisms work in parallel to ensure the robust and smooth progression of tissue closure.

## 8. Conclusions and Perspectives

This review synthesizes a series of key studies that have shed light on the molecular and cellular mechanisms of tissue closure across different developmental stages in *Drosophila*. Tissue closure is a complex process emerging from collective cell behaviors, and it leads to the formation of various delicate 3D hollow structures. Since such events occur mainly in vivo and it is challenging to replicate them in vitro, they are poorly understood. The simplicity and manipulability of the *Drosophila* make it ideal for studying complex processes such as tissue closure, allowing us to unravel its molecular and mechanical mechanisms. Moreover, due to its profound evolutionary conservation in critical biological processes, research in *Drosophila* provides valuable insights into the development of diverse species, including humans.

Despite significant progress in understanding tissue closure in *Drosophila*, several questions and limitations remain: (1) The mechanisms by which epithelial cells recognize boundary edges to coordinate cell movements and interactions for closure still puzzle scientists. (2) The integration of specific signaling pathways with mechanical forces to guide tissue morphogenesis is a vibrant area of ongoing research. (3) While the influence of tissue geometry and mechanical properties on closure processes has been studied, certain measurement techniques are invasive or limited in resolution, presenting challenges in capturing subtle dynamic changes in cells and tissues during closure. The combination of omics analysis with single-molecule and single-cell analysis will bring new discoveries in this field. (4) Tissue closure exhibits considerable plasticity and diversity across different organisms and developmental stages. Although *Drosophila* is an extremely useful model system, its differences from vertebrates in anatomical structure and physiological functions necessitate comparing findings from *Drosophila* with those from other model organisms, such as *zebrafish* and *mice*, to gain deeper insights into the evolutionary conservation and diversity of these fundamental developmental processes. Future research will require interdisciplinary collaboration, harnessing advanced technologies and innovative methodologies to overcome existing challenges in this field. In summary, upcoming studies will build on the foundation established by current research, enhancing our understanding of the complex and dynamic mechanisms driving tissue closure during development and tissue repair.

## Figures and Tables

**Table 1:** Signaling pathways in tissue closure.

Signaling Pathways	Embryogenesis			Metamorphosis		Oogenesis		Pathophysiology
	Dorsal Closure	Ventral Closure	Dorsal Vessel Closure	Thoracic Closure	Wing Vein Closure	Follicle Cell Sheet Closure	Dorsal Appendage Formation	Wound Healing
DPP	[13,16,19,28]	[30]	[40]	[50,51]	[49,58]	[67,68]	[71,72]	[76]
EGF	[13]	[30]	[40]	N/D	[49,58]	[65,68]	[71,72]	[24]
FGF	N/D	N/D	[36,39,40]	N/D	N/D	[69]	N/D	[89]
Hedgehog	N/D	N/D	[40]	N/D	[49,58]	[67]	N/D	N/D
Hippo	[19]	N/D	N/D	N/D	N/D	[67]	N/D	[27,76,93]
JAK/STAT	N/D	N/D	N/D	N/D	N/D	[62,67]	N/D	[89]
JNK	[13,16,19,22,28,29,52]	N/D	[36,40]	[51,52]	N/D	N/D	[72]	[73,76,89,91]
Notch	[13]	N/D	[40]	N/D	[49,58]	N/D	N/D	N/D
Pvr	[29]	N/D	[40]	[29,52]	N/D	[67]	N/D	[85]
Toll	N/D	[30]	[39]	N/D	N/D	N/D	N/D	[73,76]
Wnt	[19,25]	N/D	[40]	[46,50]	[58]	[67]	N/D	[76]

N/D: Not determined.
